# Early-Onset Negative Energy Balance in Transition Dairy Cows Increases the Incidence of Retained Fetal Membranes

**DOI:** 10.3390/ani16020229

**Published:** 2026-01-13

**Authors:** Zhihong Zhang, Shanshan Guo, Jianhao Yang, Xinfeng Hou, Xia Zhang, Huifeng Liu, Tao Liu, Yaping Jin

**Affiliations:** 1 College of Veterinary Medicine, Northwest A&F University, Yangling, Xianyang 712100, China; zhangzhihong0613@163.com (Z.Z.);; 2 Key Laboratory of Animal Biotechnology, Ministry of Agriculture and Rural Affairs, Northwest A&F University, Yangling, Xianyang 712100, China

**Keywords:** retained fetal membranes, negative energy balance, lipid mobilization, muscle mobilization, periparturient dairy cows

## Abstract

This study investigated the metabolic mechanisms driving physiological functional remodeling in RFM by analyzing plasma biochemical parameters and metabolomic profiles at key peripartum timepoints (21 and 7 d prepartum and 4 h postpartum), integrated with placental and fetal membrane metabolic characteristics. The results revealed that RFM cows exhibited significant negative energy balance (NEB) as early as 21 days before parturition, characterized by elevated plasma levels of non-esterified fatty acids, β-hydroxybutyrate, and malondialdehyde, alongside reduced activity of antioxidant enzymes (GSH-Px, CAT) (*p* ≤ 0.05). Metabolomic analysis demonstrated persistent lipid metabolism dysregulation, amino acid imbalance, and nucleotide metabolism disturbances in RFM cows from 21 days prepartum to 4 h postpartum, indicating premature mobilization of adipose and muscle tissues. Further metabolomic analyses of the placenta and fetal membranes confirmed that metabolic dysfunction compromises energy supply during parturition, adversely affecting immune homeostasis and extracellular matrix degradation in the placenta and fetal membranes of RFM dairy cows. These physiological dysfunctions have the potential to impede the timely expulsion of fetal membranes after calving. In conclusion, RFM is closely associated with early-onset metabolic dysfunction during the periparturient period, where insufficient energy supply due to NEB, oxidative stress, and immune-endocrine disruptions collectively impair normal fetal membrane detachment.

## Simple Summary

Retained fetal membrane (RFM), a significant reproductive disorder in dairy cattle, adversely affects both animal health and production efficiency. It is concerning that the underlying pathogenesis of RFM remains incompletely elucidated. In this trial, a longitudinal approach was employed to investigate the metabolic dysregulation associated with RFM by analyzing temporal changes in plasmic biochemical parameters and metabolomic profiles at key periparturient time points (21 and 7 d prepartum and 4 h postpartum) in primiparous RFM dairy cows. By integrating these findings with metabolomic data from placental tissues and the fetal membranes themselves, we seek to interpret the physiological functional alterations occurring in the prepartum period of RFM cows from a metabolic perspective. The ultimate goals are to establish high-specificity predictive biomarkers for RFM and to advance mechanistic understanding of RFM pathogenesis through metabolic pathway analysis.

## 1. Introduction

Retained fetal membrane (RFM) refers to a pathological condition in dairy cows where, after calving, the fetal membranes are not completely expelled within 12 to 24 h, leading to their partial retention in the uterus [1]. In intensive dairy farming systems, RFM incidence exceeds 5% [2,3,4]. The decomposition and putrefaction of the retained placental tissues in the uterus of RFM dairy cows could promote the proliferation of pathogenic microorganisms in the reproductive tract, leading to secondary clinical endometritis [5]. Additionally, RFM significantly increases the occurrence of other postpartum disorders, including mastitis, ketosis, and postpartum lameness [6], posing significant threats to cow health and causing substantial economic losses [2,7,8].

The bovine placenta is a cotyledonary, mixed-type structure formed by the interdigitation of fetal chorionic villi and maternal endometrial caruncles [9]. The inter-placental region and fetal membrane-endometrium interface contain numerous binucleate and trinucleate cells embedded in a collagen and fibrillin-rich matrix, creating a highly adherent placental-uterine connection [10,11,12]. This structural organization contributes to the high incidence of RFM in dairy cows. Normal placental detachment requires moderate activation of placental innate immunity, which will further induce the apoptosis of multinucleated cells and degradation of the collagenous matrix [13]. Disruptions in immunological, metabolic, or biochemical functions in the uterus or placental tissues impair this maturation process. Consequently, fetal membranes remain adhered to the uterine wall, preventing timely expulsion and leading to RFM [5,14].

Although RFM is primarily classified as a reproductive disorder, some studies suggest it should also be considered a metabolic disease due to its strong association with periparturient metabolic dysregulation. RFM cows consistently exhibit severe metabolic dysfunction during the transition period, including negative energy balance (NEB) [15,16], oxidative stress and hypocalcemia [17]. These metabolic disturbances disrupt immune homeostasis, interfering with placental maturation and detachment, ultimately leading to RFM [18,19,20]. To avoid the influence of previous parities on RFM in dairy cows, this study used periparturient primiparous cows as experimental animals. This study aims to elucidate the physiological function remodeling of RFM dairy cows from a metabolic perspective by analyzing key biochemical indicators (e.g., lipid/protein mobilization, oxidative stress) and plasma metabolites at critical perinatal time points (−21 d, −7 d, and +4 h relative to calving) to identify potential diagnostic markers. By integrating metabolomic data from the placenta and fetal membranes, a comprehensive metabolic regulatory network underlying RFM is constructed, which facilitates in-depth exploration of the disease etiology.

## 2. Materials and Methods

### 2.1. Animals, Housing and Experimental Design

The use of animals and experimental protocols in this study was approved by the Animal Care and Use Committee of the College of Veterinary Medicine at Northwest A&F University (Ethical Approval number: DY2024076). The trial involved 100 healthy, primiparous, age-matched (23.9 ± 0.28 months) periparturient Holstein dairy cows (bought from Junlebao Dairy Co., Ltd., Zhangjiakou, China). All cows were housed under identical confined conditions, receiving a standardized diet (Table 1) and feeding management. Uniform husbandry practices were maintained, including thrice-daily scheduled feedings with ad libitum access to feed and water throughout the trial.

Cows exhibiting abnormal calving (e.g., preterm delivery, dystocia, twins, or stillbirth) or diagnosed diseases (e.g., mastitis, postpartum paresis, ketosis, or pneumonia) were excluded. RFM was defined as the failure to expel fetal membranes within 12 h postpartum. After excluding cows with significant deviations from expected calving dates, anomalies, or illnesses, 9 cows were assigned to the RFM group, while 9 normal calving cows served as controls (CON).

### 2.2. Phenotypic Indicators of Dairy Cows

#### 2.2.1. Body Weight and Dry Matter Intake (DMI)

The body weight of dairy cows was measured between 8:00 and 9:00 AM on day 7 before calving using a calibrated livestock scale. The DMI was recorded as total daily dry matter intake (kg/d), with data logged against individual cow ear tag numbers.

#### 2.2.2. Body Temperature

In this trial, an infrared auricular thermometer was used to monitor external ear canal temperature twice daily (08:00–09:00 and 16:00–17:00) during the 7-day prepartum period. Daily mean temperature was calculated from the two measurements.

#### 2.2.3. Respiratory Rate

Flank movements were counted over 10-min intervals at three daily timepoints (09:00–09:30, 12:00–12:30, and 17:00–17:30) for 7 days pre-calving. The triplicate measurements were averaged to determine daily respiratory rate.

#### 2.2.4. Step Count

A validated pedometer system continuously recorded locomotor activity (steps/day) throughout the final 7 prepartum days.

### 2.3. Plasmic Biochemical Analyses and Metabolomic Profiling

#### 2.3.1. Blood Sampling Protocol

Peripheral blood samples (20 mL) were collected from dairy cows via the caudal vein during morning feeding at 3 time points: 21 days (n_CON_ = 5, n_RFM_ = 8) and 7 days (n_CON_ = 4, n_RFM_ = 4) before calving and at 4 h post-calving (n_CON_ = 7, n_RFM_ = 9). Heparin sodium anticoagulant tubes were used for collection. Samples were centrifuged at 4 °C and 2000 rpm for 10 min to isolate plasma, which was subsequently stored at −80 °C until analysis.

#### 2.3.2. Plasmic Biochemical Analyses

In this experiment, we analyzed plasma glucose (GLU), non-esterified fatty acid (NEFA), β-hydroxybutyric acid (βHB), and lactic acid (LAC) levels, as well as plasma lactic dehydrogenase (LDH) activity, to assess NEB and lipid metabolism in dairy cows. The activities of aspartate aminotransferase (AST), alanine aminotransferase (ALT), creatine kinase (CK), and concentrations of blood urea nitrogen (BUN) and total bilirubin (TBIL) were analyzed to evaluate the severity of muscle tissue mobilization and hepatic injury, as these biomarkers exhibit correlations with such physiological changes.

Both NEB-related lipid metabolism and muscle tissue mobilization can induce oxidative stress in dairy cows. The oxidative stress markers measured in this study included superoxide dismutase (SOD), glutathione peroxidase (GSH-Px), and catalase (CAT) activities, as well as malondialdehyde (MDA) concentration. All plasma biochemical indices were detected using research-grade assay kits provided by Genebest (Suzhou) Biotechnology Co., Ltd. (Suzhou, Jiangsu, China), with strict adherence to the manufacturer’s protocols. Additionally, plasma calcium ion concentration was determined via inductively coupled plasma mass spectrometry (ICP-MS).

#### 2.3.3. Plasmic Metabolome

A non-targeted metabolomics approach (liquid chromatography-mass spectrometry, LC-MS) was adopted to analyze the plasmic metabolomic profiling. Following quality control, normalization, and standardization processing of metabolomic data, the relative abundances of various small-molecule metabolites were determined (Table S1). Partial Least Squares Discriminant Analysis (PLS-DA) was employed to examine the distribution patterns of metabolomic samples, elucidating the systemic impact of RFM on dairy cow metabolic function. Differential metabolites (DMs) were screened (*t*-test, *p* ≤ 0.05, VIP-OPLS-DA > 1.0), and a clustering heatmap of Z-score-transformed DMs was generated. Subsequently, Kyoto Encyclopedia of Genes and Genomes (KEGG) enrichment analysis was performed based on the identified DMs to delineate the characteristic metabolic alterations in RFM-affected dairy cows during critical prepartum time points.

### 2.4. Metabolomics Analysis of Placenta and Fetal Membrane

After calving, placental (n_CON_ = 6, n_RFM_ = 3) and fetal membrane (n_CON_ = 7, n_RFM_ = 5) tissue samples were promptly collected from the RFM and CON dairy cows, respectively. The samples were rinsed three times with 4 °C PBS buffer, then placed into cryovials. Then, the cryovials were flash-frozen in liquid nitrogen and subsequently transferred to a −80 °C freezer for storage, pending further metabolomic analysis by using the LC-MS method. Then, the processed metabolomic data (QC, normalized, standardized, Table S1) underwent PLS-DA to assess the metabolic impact of RFM in the placenta and fetal membrane of dairy cows. A volcano plot was generated to visually represent the relative abundances of various metabolites and highlight the DMs (*t*-test, *p* ≤ 0.05, VIP-OPLS-DA > 1.0). DMs were visualized via Z-score heatmap and analyzed through KEGG enrichment to reveal prepartum metabolic disruptions in the placenta and fetal membrane of RFM dairy cows.

### 2.5. Statistical Analysis

The statistical analysis of the experimental data was performed using SPSS 20.0. Differences in dairy cow phenotypic traits, as well as plasma biochemical markers related to lipid mobilization, protein mobilization, and oxidative stress, were analyzed using Levene’s test for homogeneity of variance and Student’s *t*-test. The screening criteria for differential metabolites in serum, placental, and fetal membrane tissue metabolomics were set at *p* ≤ 0.05 and VIP-OPLS-DA > 1.0. All the aforementioned experimental data were presented as mean ± standard deviation, with statistical significance set at *p* ≤ 0.05.

## 3. Results

### 3.1. Phenotypic Characteristic of RFM Dairy Cow

**Figure 1:**
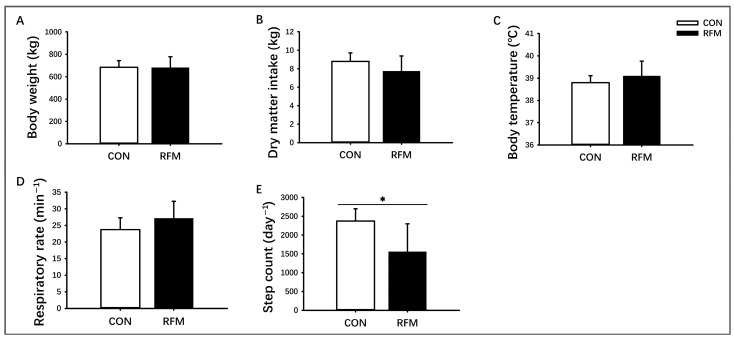
The physiological characterization of retained fetal membranes (RFM) and normal calving (CON) dairy cows at 7 days before calving. (**A**) Body weight, (**B**) Dry matter intake, (**C**) Body temperature, (**D**) Respiratory rate, and (**E**) Daily step count. The data was shown as mean ± SD, * *p* ≤ 0.05.

As depicted in Figure 1, at 7 days pre-calving, there was no significant difference in body weight between dairy cows with RFM (627 ± 93 kg) and the CON (649 ± 46 kg). However, RFM cows exhibited a higher degree of individual variation, with more animals having either abnormally high or low body weights (Figure 1A). RFM had no significant effect on pre-calving DMI (Figure 1B), body temperature (Figure 1C), or respiratory rate (Figure 1D) at 7 days before calving. In contrast, the average daily step count of RFM cows (1526 ± 727 steps) was significantly lower than that of CON cows (2364 ± 409 steps) (*p* ≤ 0.05; Figure 1E). Behavioral observations further revealed that some RFM cows displayed prolonged recumbency, reduced activity, and a tendency to isolate themselves from the herd at 7 days before calving.

### 3.2. Plasmic Biochemical Indicators of RFM Dairy Cows

#### 3.2.1. Lipid Mobilization

**Figure 2:**
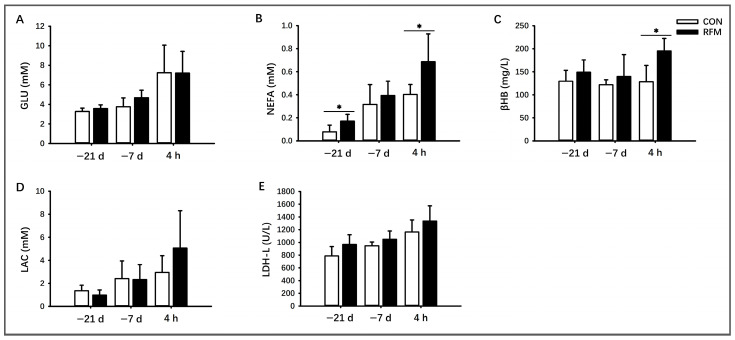
Plasmic biochemical indicators related to negative energy balance from RFM and CON dairy cows at 21 days and 7 days prepartum and 4 h postpartum. (**A**–**E**) Energy balance indicators: plasma concentrations of (**A**) GLU, (**B**) NEFA, (**C**) βHB, (**D**) LAC, and (**E**) LDH-L activity. All experimental data was shown as mean ± SD, * *p* ≤ 0.05.

As shown in Figure 2, there were certain differences in plasmic energy balance-related metabolic indicators (GLU, NEFA, βHB, LAC, and LDH-L) between periparturient RFM and CON cows. The experimental results demonstrated that the plasma NEFA concentration in RFM cows was significantly elevated (*p* ≤ 0.05) at 21 days before calving. At 4 h postpartum, both the lipid mobilization product NEFA (Figure 2B) and βHB levels (Figure 2C) in RFM cows showed a significant increase (*p* ≤ 0.05). From a temporal perspective, from 21 days prepartum to 4 h postpartum, the plasma levels of lipid mobilization product NEFA (Figure 2B) and LAC (Figure 2D) in cows rose significantly, indicating a progressive exacerbation of NEB during the periparturient period. Moreover, RFM cows exhibited more severe NEB compared to normal-calving cows.

#### 3.2.2. Muscle Tissue Mobilization

**Figure 3:**
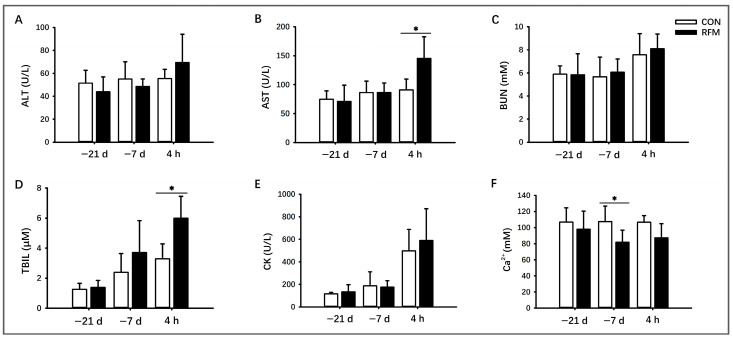
Plasmic biochemical indicators related to organic protein mobilization from RFM and CON dairy cows at 21 days and 7 days prepartum and 4 h postpartum. (**A**–**F**) Tissue mobilization and calcium status: plasma activities of (**A**) alanine aminotransferase (ALT), (**B**) aspartate aminotransferase (AST), and (**C**) creatine kinase (CK). Plasma concentrations of (**D**) total bilirubin (TBIL), (**E**) blood urea nitrogen (BUN), and (**F**) calcium ion (Ca^2+^). All experimental data was shown as mean ± SD, * *p* ≤ 0.05.

Due to more severe NEB and lipid mobilization during the transition period in dairy cows with RFM, this study further investigated the effects of RFM on serum indicators related to muscle tissue mobilization (Figure 3). The experimental results demonstrated that plasmic AST activity (Figure 3B) and TBIL levels (Figure 3D) were significantly elevated (*p* ≤ 0.05) in RFM cows at 4 h postpartum. In contrast, ALT activity (Figure 3A), BUN levels (Figure 3C), and CK activity (Figure 3E) showed no significant differences at any time point during the transition period. From a temporal perspective, plasmic AST and CK activities, as well as BUN and TBIL levels, were significantly higher at 4 h postpartum compared to those at 21 days prepartum. This indicates greater mobilization of muscle tissue in cows just after parturition. Furthermore, the results suggest that the liver dysfunction induced by tissue mobilization was more severe in RFM cows, specifically at 4 h postpartum.

#### 3.2.3. Plasma Ca^2+^ Dynamics

Simultaneously, plasma Ca^2+^ concentration was significantly lower (*p* ≤ 0.05) in RFM cows compared to normally calving cows at 7 days prepartum. At 4 h postpartum, plasma Ca^2+^ in RFM cows also showed a declining trend (0.05 ≤ *p* ≤ 0.10). Critically, the plasma Ca^2+^ of RFM cows at both 7 days prepartum and 4 h postpartum was below the hypocalcemia threshold (88.2 mg/L) (Figure 3F). This indicates that RFM cows demonstrated a higher incidence of hypocalcemia.

#### 3.2.4. Antioxidant Ability

**Figure 4:**
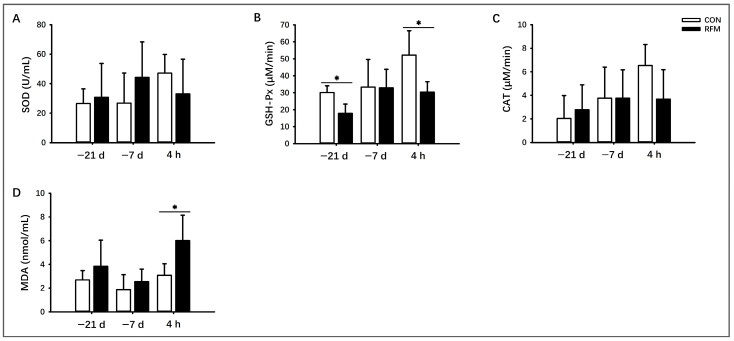
Plasmic biochemical indicators related to antioxidant ability from RFM and CON dairy cows at 21 days and 7 days prepartum and 4 h postpartum. (**A**–**D**) Antioxidant ability markers: Activities of (**A**) superoxide dismutase (SOD), (**B**) glutathione peroxidase (GSH-Px), (**C**) catalase (CAT), and (**D**) malondialdehyde (MDA) concentration. All experimental data was shown as mean ± SD, * *p* ≤ 0.05.

We analyzed the effects of RFM on the activities of SOD, GSH-Px, and CAT, and the concentration of MDA in bovine plasma. The experimental results showed that plasmic GSH-Px activity of RFM cows was significantly decreased (*p* ≤ 0.05) at 21 days before calving and 4 h after calving (Figure 4B). RFM cow’s CAT activity also exhibited a declining trend (0.05 ≤ *p* ≤ 0.10) at 4 h postpartum (Figure 4C). Plasma MDA levels were also significantly elevated (*p* ≤ 0.05) at 4 h postpartum, with a similar upward trend observed at 21 days prepartum (Figure 4D). These results indicated that RFM cows experienced more severe oxidative stress during the periparturient period, likely due to enhanced lipid and muscle tissue mobilization compared to normal cows.

### 3.3. Plasma Metabolome Alteration of RFM Dairy Cow During the Peripartum Period

**Figure 5:**
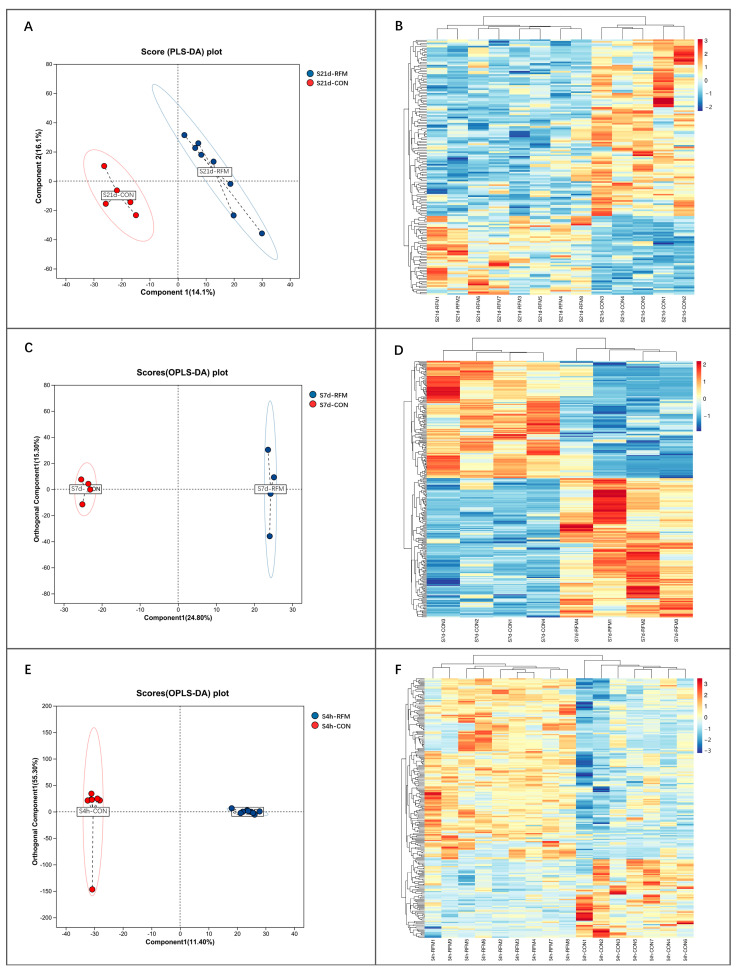
Early-onset and progressive prepartum plasma metabolomic profiling from RFM versus CON dairy cows at 21 days and 7 days prepartum and 4 h postpartum. (**A**,**B**) Metabolomic alterations at −21 d: (**A**) Partial least squares discriminant analysis (PLS-DA) score plot; (**B**) Hierarchical cluster analysis of 165 differential metabolites; (**C**,**D**) Metabolomic alterations at −7 d: (**C**) PLS-DA score plot; (**D**) Hierarchical cluster analysis of 267 differential metabolites; (**E**,**F**) Metabolomic alterations at 4 h: (**E**) PLS-DA score plot; (**F**) Hierarchical cluster analysis of 228 differential metabolites.

PLS-DA of the plasma metabolome at 21 and 7 days prepartum and 4 h postpartum revealed a significant difference in the plasma small-molecule metabolite profile of RFM cows compared to the cows experiencing normal calving (Figure 5A,C,E). Subsequent analysis identified a total of 165 DMs between the RFM and CON groups at 21 days pre-calving (115 decreased DMs, and 50 increased DMs; *p* < 0.05). 267 DMs were identified in the plasma of RFM cows at 7 days pre-calving (123 decreased DMs, and 144 increased DMs; *p* < 0.05). 228 DMs were identified in the plasma of RFM cows at 4 h after calving (69 decreased DMs, and 159 increased DMs; *p* < 0.05). Furthermore, cluster analysis based on these DMs further indicated a distinct separation in the plasma metabolite composition between RFM cows and CON cows at 21 and 7 days pre-calving and 4 h after calving (Figure 5B,D,F).

**Figure 6:**
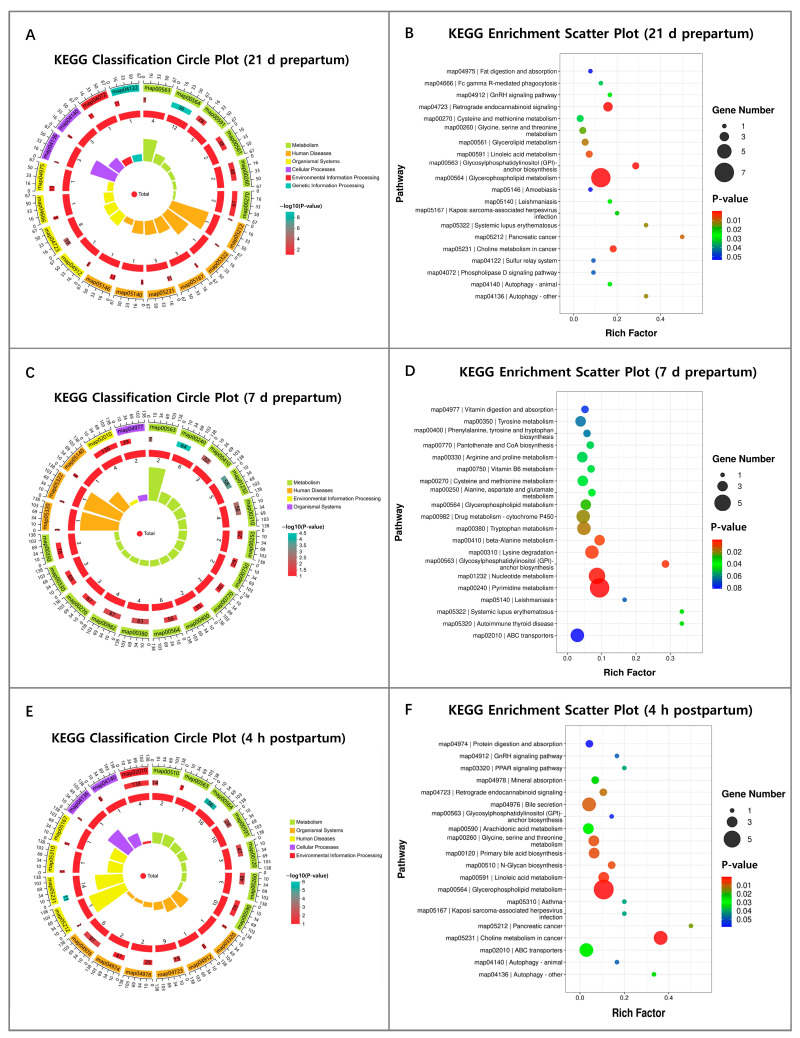
KEGG pathway enrichment analysis of plasma differential metabolites from RFM versus CON dairy cows at 21 days and 7 days prepartum and 4 h postpartum. (**A**,**C**,**E**) Classification circle plots of enriched KEGG pathways for plasma differential metabolites at (**A**) 21 days prepartum, (**C**) 7 days prepartum, and (**E**) 4 h postpartum. (**B**,**D**,**F**) Bubble plots showing the top 20 significantly enriched KEGG pathways at (**B**) 21 days prepartum, (**D**) 7 days prepartum, and (**F**) 4 h postpartum. The size of the bubble represents the number of metabolites, and the color represents the *p*-value significance.

Further KEGG enrichment analysis demonstrated that the differential plasma metabolites at 21 days prepartum were enriched in signaling pathways related to metabolic processes, human disease development, biological systems, cellular processes, environmental adaptation, and genetic information processing (*p* < 0.05; Figure 6A). A large proportion of signaling pathways were related to nutrient metabolism and immune function, indicating that RFM cows exhibit characteristics of metabolic and immune dysfunction as early as 21 days prepartum. Among the top 20 significantly enriched KEGG pathways, 8 were related to lipid, amino acid, and vitamin metabolism, including fat digestion and absorption, glycerophospholipid metabolism, glycosylphosphatidylinositol anchor (GPI-anchor) biosynthesis, linoleic acid metabolism, glycerolipid metabolism, phospholipase D signaling pathway, glycine, serine, and threonine metabolism, cysteine and methionine metabolism, and GnRH signaling pathway (*p* < 0.05; Figure 6B).

KEGG pathway enrichment analysis at 7 days prepartum showed the top 20 significantly enriched KEGG pathways mainly associated with metabolic processes (*p* < 0.05; Figure 6C). KEGG pathway enrichment analysis at 7 days prepartum revealed significant alterations in the activity of several amino acid metabolism-related pathways, including ABC transporters, lysine degradation, β-alanine metabolism, tryptophan metabolism, alanine, aspartate and glutamate metabolism, cysteine and methionine metabolism, arginine and proline metabolism, phenylalanine, tyrosine and tryptophan biosynthesis, and tyrosine metabolism. Concurrently, significant enrichment was also observed in lipid metabolism-associated pathways, including glycerophospholipid metabolism and GPI-anchor biosynthesis. Furthermore, pathways involved in nucleic acid metabolism were significantly enriched (pyrimidine metabolism and nucleotide metabolism) (*p* < 0.05; Figure 6D).

KEGG pathway enrichment analysis of differential plasma metabolites at 4 h postpartum also revealed that the DMs were primarily associated with nutrient metabolism (*p* < 0.05; Figure 6E). The differential metabolites in RFM cow plasma were closely linked to lipid metabolism pathways, including glycerophospholipid metabolism, linoleic acid metabolism, primary bile acid biosynthesis, and bile secretion. Additionally, alterations were also observed in amino acid metabolism-related pathways, such as ABC transporters, protein digestion and absorption, and glycine, serine, and threonine metabolism. Furthermore, vitamin and mineral metabolism-related pathways, including choline metabolism in cancer and mineral absorption, were also significantly affected. In addition, the significantly enriched signaling pathways also included the GnRH signaling pathway, which is critical for reproductive hormone regulation (*p* < 0.05; Figure 6F). These results indicate that early metabolic imbalance is an important contributing factor to the development of RFM.

**Figure 7:**
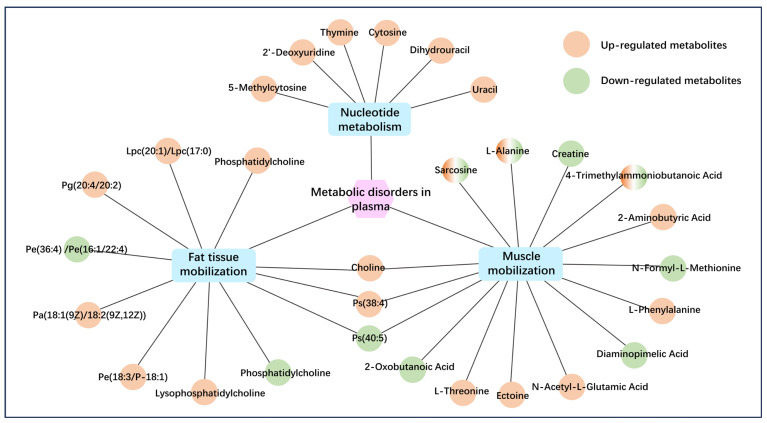
The correlation between differential metabolites and metabolic dysfunction in RFM dairy cows’ plasma. Phosphatidylcholine (Gpcho) included Gpcho (22:6/20:2), Gpcho (22:5/16:1), Gpcho (20:4/18:0), Gpcho (20:4/15:0), Gpcho (20:1/20:4), Gpcho (20:1/18:4), Gpcho (20:0/18:2), Gpcho (18:3/22:4), and Gpcho (16:0/15:0). Lysophosphatidylcholine (Lysopc) included Lysopc (P-18:0/0:0), Lysopc (18:1(11Z)/0:0), Lysopc (15:0), and Lysopc (14:1(9Z)/0:0). Phosphatidylcholine (Pc) included Pc (34:1) and Pc (14:0/P-18:1(9Z)).

**Figure 8:**
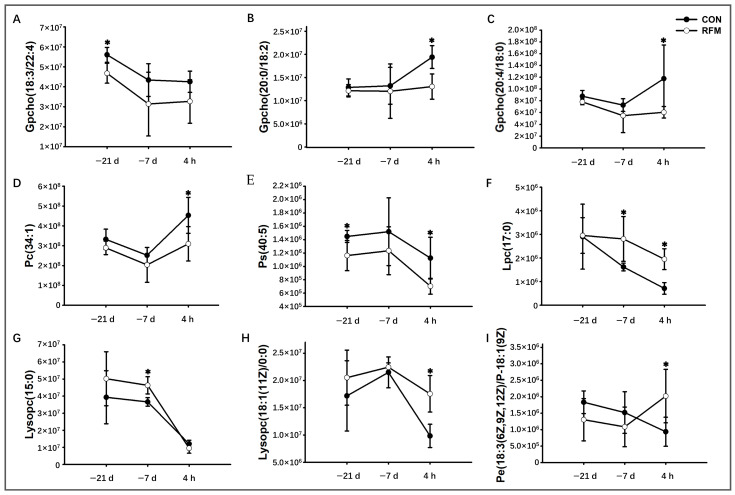
The longitudinal dynamic changes in lipid mobilization-related key plasma differential metabolites throughout the periparturient period of RFM dairy cows. (**A**) Gpcho (18:3/22:4), (**B**) Gpcho (20:0/18:2), (**C**) Gpcho (20:4/18:0), (**D**) Pc (34:1), (**E**) Ps (40:5), (**F**) Lpc (17:0), (**G**) LysoPC (15:0), (**H**) LysoPC (18:1(11Z)/0:0), and (**I**) Pe (18:3/P-18:1). Data are presented as mean ± SD. * *p* < 0.05 indicates statistical significance.

At 21 and 7 days prepartum and 4 h postpartum, plasma metabolomics revealed that RFM dairy cows underwent metabolic remodeling related to adipose and muscle tissue mobilization, as well as nucleotide metabolism, indicating potential metabolic dysregulation during the prepartum period (Figure 7). The RFM dairy cows exhibited marked variations in plasma small-molecule metabolites associated with adipose tissue mobilization, with lipid metabolite dynamics directly tied to membrane remodeling and tissue mobilization intensity. As illustrated in Figure 8, the dynamic changes in key lipid metabolites during the periparturient period reveal that the levels of phosphatidylcholine (18:3/22:4) in RFM dairy cows at 21 days prepartum, as well as plasma phosphatidylcholines (Gpcho (20:4/18:0), Gpcho (20:0/18:2), Gpcho (18:3/22:4), Pc(34:1), etc.) and the phosphatidylcholine-derived phosphatidylserine (40:5) at 4 h postpartum, were significantly lower than those in CON cows. These phosphatidylcholines are primarily synthesized by the liver for the construction of cellular membrane systems, which means that RFM cows exhibit dysregulated remodeling of membrane phospholipids. By 7 days prepartum and 4 h postpartum, lysophosphatidyl choline (LPC (17:0) and LysoPC (15:0)) were markedly elevated, reflecting exacerbated adipose/muscle tissue mobilization and cellular breakdown.

**Figure 9:**
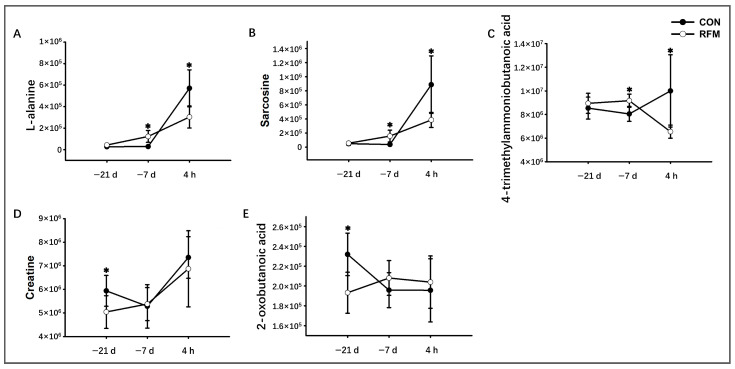
The longitudinal dynamic changes in plasmic protein mobilization-related key plasma differential metabolites throughout the periparturient period of RFM dairy cows. (**A**) L-alanine, (**B**) sarcosine, (**C**) 4-trimethylammoniobutanoic acid, (**D**) creatine, and (**E**) 2-oxobutanoic acid. Data are presented as mean ± SD. * *p* < 0.05 indicates statistical significance.

The plasma levels of muscle mobilization-related metabolites were significantly altered in periparturient RFM dairy cows. As shown in Figure 9, RFM dairy cows showed reduced concentrations of important rapid-energy suppliers (creatine and 2-oxobutyrate) in plasma at 21 days prepartum, suggesting earlier energy insufficiency as a driver of premature tissue mobilization. By 7 days prepartum, elevated plasmic L-alanine and sarcosine levels in RFM dairy cows confirmed intensified muscle catabolism. However, these metabolites decreased below control levels by 4 h postpartum, demonstrating insufficient energy reserves due to prolonged NEB, which likely compromises parturition-related energy supply and elevates RFM risk while impairing early lactation performance.

**Figure 10:**
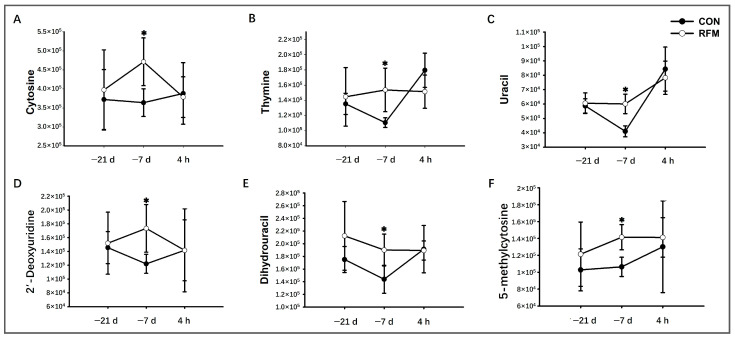
The longitudinal dynamic changes in plasmic nucleotide metabolism-related key plasma differential metabolites throughout the periparturient period of RFM dairy cows. (**A**) cytosine, (**B**) thymine, (**C**) uracil, (**D**) dihydrouracil, (**E**) 2′-deoxyuridine, and (**F**) 5-methylcytosine. Data are presented as mean ± SD. * *p* < 0.05 indicates statistical significance.

During the periparturient period, notable alterations were also detected in nucleotide metabolism within the plasma of RFM dairy cows. At 21 days prepartum, although the plasma concentrations of cytosine, thymine, 2′-deoxyuridine, and 5-methylcytosine in RFM dairy cows showed no statistical significance, they exhibited an increasing trend, suggesting early signs of tissue mobilization. By 7 days prepartum, the levels of cytosine, thymine, uracil, dihydrouracil, 2′-deoxyuridine, and 5-methylcytosine were significantly elevated, confirming that intensified NEB had substantially aggravated tissue mobilization. However, at 4 h postpartum, no differences were observed between RFM and CON dairy cows, indicating that their tissue mobilization levels had converged after parturition (Figure 10). Above all, the plasma metabolomic profile revealed pre-existing dysregulation of lipid, amino acid, and nucleotide metabolism in RFM dairy cows as early as 21 days before calving. Such metabolic imbalances likely impair nutrient availability during parturition, ultimately disrupting the physiological detachment of fetal membranes after delivery.

### 3.4. Metabolomic Analysis of RFM Dairy Cows’ Placenta and Fetal Membrane

#### 3.4.1. Metabolomic Analysis of Placenta

**Figure 11:**
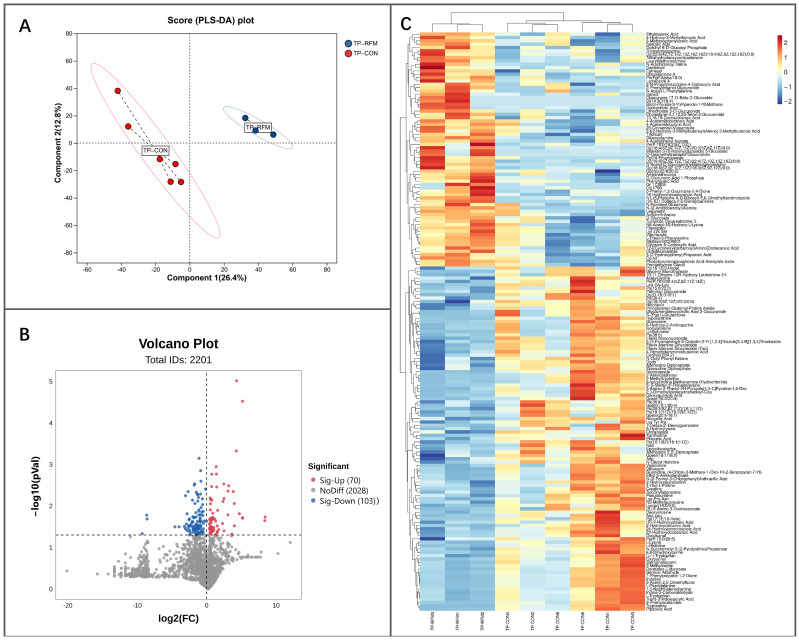
The overall metabolic composition of newly delivered placenta from RFM versus CON dairy cows. (**A**) PLS-DA score plot; (**B**) Volcano plot identifying significantly upregulated and downregulated metabolites (*p* ≤ 0.05); (**C**) Hierarchical cluster heatmap of 173 placental DMs.

The composition of small-molecule metabolites in the placenta of RFM cows exhibited certain alterations in comparison to the normal-delivery dairy cows. PLS-DA results indicated a discernible difference in the placental metabolic profile between RFM and CON cows (Figure 11A). Compared to the CON group, 173 differentially abundant metabolites (DMs) were identified in RFM cow placentas (*t*-test, *p* ≤ 0.05), with 70 metabolites showing significantly increased concentrations and 103 metabolites demonstrating significantly decreased concentrations (Figure 11B). Cluster analysis based on the differential metabolite set yielded consistent results with the PLS-DA, confirming that the metabolic functional state of RFM cows’ placenta underwent significant alterations (Figure 11C).

**Figure 12:**
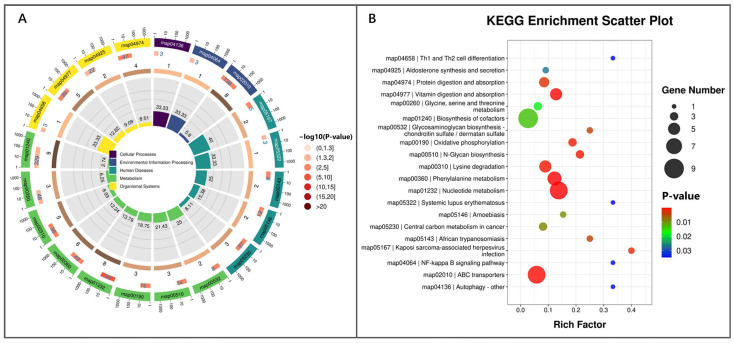
KEGG enrichment analysis of placental differential metabolites screened from RFM versus CON dairy cows. (**A**,**B**) KEGG pathway enrichment analysis visualized by (**A**) circle plot and (**B**) scatter plot.

RFM could systematically affect the metabolism in the placenta of RFM dairy cows. The circular diagram of KEGG enrichment analysis revealed that among the 20 significantly enriched KEGG pathways, most were related to immune regulation and nutrient metabolism (Figure 12A). In RFM dairy cows’ placenta, the systemic lupus erythematosus pathway, a key pathway linked to immune tolerance, showed significant alterations. Additionally, pathways associated with immune function regulation, including Th1 and Th2 cell differentiation, the NF-κB signaling pathway, and autophagy, were also modulated, indicating immune dysfunction in placental tissues of RFM cows. Compared to the CON cows, RFM cows exhibited marked changes in pathways related to pathogenic infections, such as Kaposi sarcoma-associated herpesvirus infection, African trypanosomiasis, and amoebiasis, suggesting a potential correlation between these infections and the high incidence of RFM.

Significant alterations in nutrient metabolism were also observed in the placenta of RFM dairy cows. Key pathways related to amino acid transport and metabolism, including ABC transporters, protein digestion and absorption, phenylalanine metabolism, lysine degradation, and glycine, serine, and threonine metabolism, were markedly affected. Additionally, nucleotide metabolism, vitamin digestion and absorption, biosynthesis of cofactors, central carbon metabolism in cancer, and oxidative phosphorylation pathways were found to influence vitamin homeostasis and energy metabolism. These findings further indicate that RFM cows are prone to more severe NEB.

Furthermore, significant alterations were observed in the N-glycan biosynthesis pathway and the glycosaminoglycan biosynthesis chondroitin sulfate/dermatan sulfate pathway, both of which adversely affect placental expulsion postpartum. Additionally, the endocrine signaling pathway, aldosterone synthesis and secretion, may also participate in regulating placental expulsion in dairy cows after calving (Figure 12B). These KEGG pathways may collectively increase the risk of RFM in postpartum cows.

#### 3.4.2. Metabolomic Analysis of Fetal Membranes

**Figure 13:**
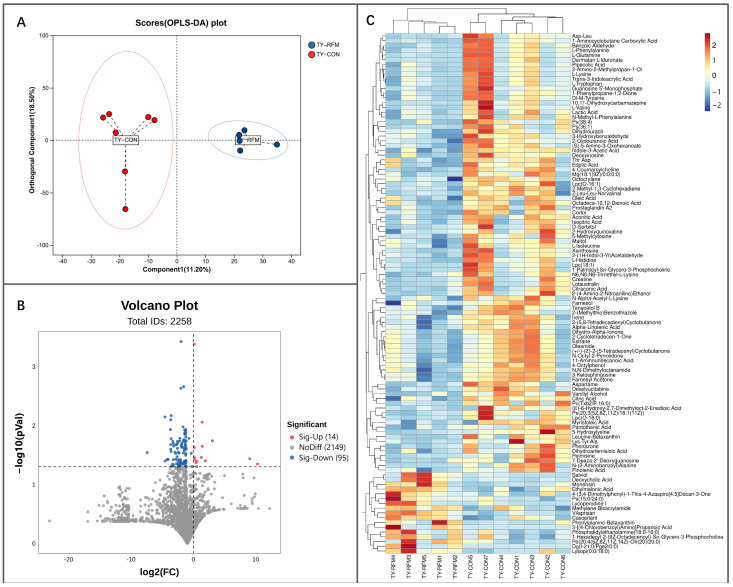
The overall metabolic composition of newly delivered fetal membranes of RFM and CON dairy cows. (**A**) PLS-DA score plot; (**B**) Volcano plot identifying significantly upregulated and downregulated metabolites (*p* ≤ 0.05); (**C**) Hierarchical cluster heatmap of 173 placental differential metabolites.

The PLS-DA results revealed distinct differences in the fetal membranes` metabolic composition of RFM compared to CON cows (Figure 13A). The volcano plot identified 109 DMs in RFM cow placentas (*t*-test, *p* ≤ 0.05), with 14 metabolites showing significantly increased and 95 metabolites exhibiting significantly decreased (Figure 13B). Cluster analysis of DMs further confirmed alterations in the metabolic function of RFM fetal membranes (Figure 13C).

**Figure 14:**
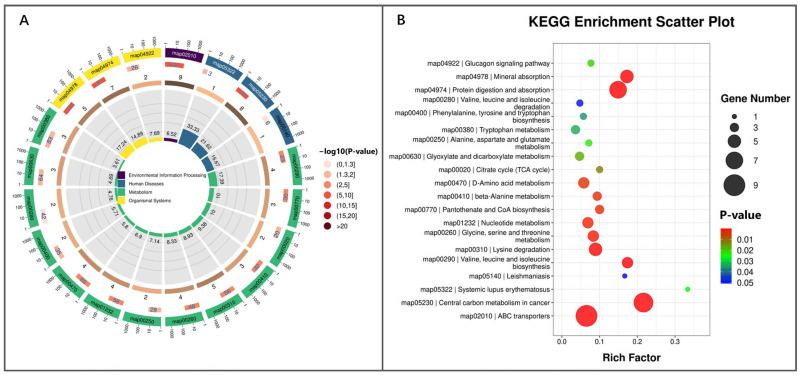
KEGG enrichment analysis of differential metabolites from fetal membranes screened with RFM versus CON dairy cows. (**A**,**B**) KEGG pathway enrichment analysis visualized by (**A**) circle plot and (**B**) scatter plot.

KEGG enrichment analysis based on the DMs revealed significant alterations in the activity of signaling pathways related to cellular processes, environmental information processing, human diseases, metabolism, and organismal systems in the fetal membranes of dairy cows with RFM. Among the top 20 most significantly different pathways, 13 were metabolism-related signaling pathways (Figure 14A). This suggests that metabolic dysfunction in fetal membranes of dairy cows represents a significant contributing factor to the elevated incidence of RFM.

As shown in Figure 14B, RFM significantly impacted the ABC transporters signaling pathway, which is involved in the transport of carbohydrates, amino acids, inorganic ions, and vitamins. The fetal membranes of RFM cows exhibited significant changes in the activity of pathways crucial for energy metabolism, namely glyoxylate and dicarboxylate metabolism, the TCA cycle, the glucagon signaling pathway, and central carbon metabolism in cancer. Concurrently, the activity status of multiple pathways related to amino acid metabolism, such as protein digestion and absorption, valine, leucine and isoleucine biosynthesis, lysine degradation, glycine, serine and threonine metabolism, β-alanine metabolism, D-amino acid metabolism, alanine, aspartate and glutamate metabolism, tryptophan metabolism, phenylalanine, tyrosine and tryptophan biosynthesis, valine, leucine and isoleucine degradation, was significantly changed in RFM fetal membrane tissue. In addition to energy metabolism and amino acid metabolism, significant changes were also observed in the nucleotide metabolism, pantothenate and CoA biosynthesis, and mineral absorption signaling pathways within the RFM cows’ fetal membrane.

#### 3.4.3. The Correlation Between DMs and Metabolic Dysfunction in Placenta and Fetal Membranes of RFM Dairy Cows

**Figure 15:**
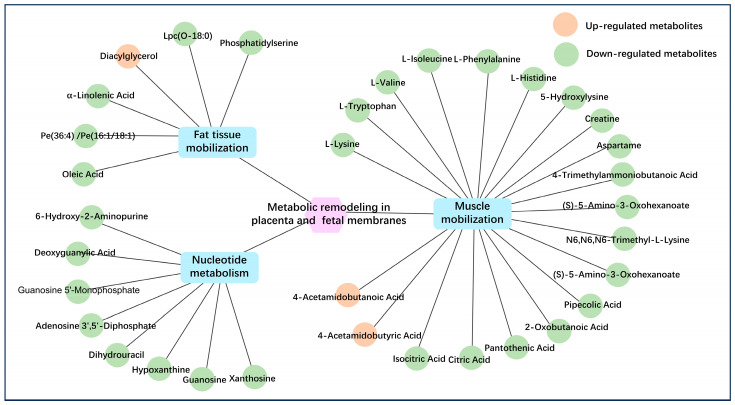
The correlation between differential metabolites and metabolic dysfunction in the placenta and fetal membranes of RFM dairy cows. Phosphatidylserine (Ps) included Ps (36:1), Ps (15:0/24:0), Ps (38:4), Ps (20:3/18:1), Ps (38:4), Ps (20:3/18:1), Ps (18:1/18:2), and Ps (15:0/22:0). Diacylglycerol (Dg) included Dg (22:6/18:4/0:0), Dg (18:4/22:4/0:0), Dg (18:4/20:3/0:0), Dg (18:4/18:3/0:0), and Dg (18:2/22:0/0:0).

Subsequently, using the significantly enriched KEGG signaling pathways and their corresponding key differential metabolites screened from placenta and fetal membranes as the foundation, we generated a correlation diagram depicting the relationships between DMs and metabolic dysfunction in RFM dairy cows. As illustrated in Figure 15, apart from several diacylglycerols (including Dg (22:6/18:4/0:0), Dg (18:4/22:4/0:0), Dg (18:4/20:3/0:0), Dg (18:4/18:3/0:0), and Dg (18:2/22:0/0:0)) associated with pathogen invasion and immune dysregulation, as well as the polyamine degradation product 4-acetamidobutyric acid, RFM cows displayed significantly reduced levels of lipid metabolites, amino acids (e.g., L-histidine, L-isoleucine, L-valine) and their derivatives, nucleotide metabolites from tissue mobilization, and TCA cycle intermediates (e.g., citrate and isocitrate). In the placental and fetal membrane tissues of RFM-affected dairy cows, these small molecular metabolites serve as fuel sources for oxidative breakdown to generate cellular energy. Their marked depletion compromises energy provision throughout the calving period, impeding the successful expulsion of fetal membranes and potentially elevating the risk of RFM development.

## 4. Discussion

Since the 1950s, genetic breeding, nutritional optimization, and intensified feeding management practices have led to sustained increases in milk yield and feed efficiency of dairy cows. However, this progress has concurrently intensified the metabolic burden on cows during lactation, significantly impacting key physiological systems including the mammary gland, reproductive tract, liver, and so on [21]. During the prepartum period, dairy cows require substantial amounts of metabolic energy and amino acids to support fetal development and the regeneration of mammary tissue, reproductive organs, and other visceral tissues. Furthermore, the inflammatory cytotoxic reactions associated with placental maturation within the uterus also impose significant demands on energy and amino acids [22]. However, periparturient cows experience crucial characteristic metabolic adaptations, notably depressed DMI and adipose tissue-specific insulin resistance [23]. Consequently, the metabolic energy, amino acids, and other nutrients consumed are insufficient to meet the physiological demands of a dairy cow during the transition period, which will further result in NEB. To compensate, dairy cows must mobilize body fat reserves and, to a lesser extent, body protein reserves to fulfill their nutritional requirements [15,16,24].

By 2–5 days prior to parturition, the bovine fetus reaches full maturity. Concurrently, placental maturation is achieved through the coordinated interplay of the uterine immune system [25,26] and extracellular matrix degradation system [27,28]. At this stage, fetal membranes detach completely from the endometrial epithelium, while the fetal-maternal placental interface separates almost entirely, with only the cotyledonary structures remaining attached. This separation triggers the onset of natural labor, during which uterine contractions compress the mature placenta and fetus to facilitate their expulsion [29]. Following fetal delivery, umbilical cord rupture reduces placental blood flow, prompting rapid atrophy of placental villi. Consequently, the size and surface area of placental cotyledons diminish sharply, allowing for their natural detachment from maternal crypts. Subsequent uterine contractions then expel the fetal membranes completely [1]. Notably, this entire physiological sequence requires substantial metabolic energy. During parturition, cows often experience severe energy deficits, necessitating the rapid mobilization of fat and muscle tissues to meet the heightened energy demands [30]. This suggests that energy metabolism disorders in prepartum dairy cows negatively impact natural parturition and hinder the smooth expulsion of fetal membranes following delivery.

Emerging research indicates that periparturient dairy cows adaptively regulate metabolism to maintain homeostasis amid environmental or physiological shifts [31]. RFM dairy cows frequently show energy metabolic dysfunction in the prepartum period [30]. In this trial, RFM cows show a declining trend in DMI, while pedometer data indicates markedly reduced activity levels at 7 days before calving. These phenotypic changes collectively exacerbate the NEB status in RFM cows and elevate the risk of metabolic disorder. The results of plasma biochemical analysis revealed that cows with RFM exhibited significantly higher plasma βHB concentrations at 21 days before calving and 4 h after calving compared to cows with normal calving. Additionally, the level of NEFA was also significantly elevated at 4 h post-calving in RFM cows. Furthermore, RFM cows showed a significant increase in plasma AST activity and TBIL level at 4 h after calving. These findings indicate that RFM cows experience a more severe state of NEB, accompanied by higher levels of lipid mobilization and liver damage. Periparturient NEB and decreased feed intake can lead to reduced Ca^2+^ absorption and a significant increase in Ca^2+^ loss in dairy cows, thereby elevating the risk of hypocalcemia [30,32]. In this study, RFM cows had a significantly lower plasma Ca^2+^ concentration at 7 days before calving. Furthermore, the average plasma Ca^2+^ concentrations in RFM cows at both 7 days before calving and 4 h after calving were below the critical threshold for subclinical hypocalcemia in cows (Ca^2+^ ≤ 88.2 mg/L). This indicates a risk of hypocalcemia in RFM dairy cows during the pre-peripartum period. Above all, RFM cows had already exhibited signs of NEB at 21 days before calving, and by 7 days before calving, they further developed hypocalcemia.

Pre-peripartum NEB in cows induces body fat mobilization, which in turn increases the incidence of subclinical ketosis and hypocalcemia [33,34]. These metabolic disorders can adversely affect cow physical performance, resulting in weakened uterine smooth muscle contractions during parturition [35], thereby increasing the risk of RFM development. Furthermore, hypocalcemia significantly reduces neutrophil counts [36] and impairs their immune phagocytic function by compromising the migration, adhesion, and phagocytic capabilities of polymorphonuclear neutrophils of dairy cows [37]. Notably, neutrophils play critical roles in phagocytosis and inflammatory activation during prepartum placental maturation and parturition. Hypocalcemia-induced neutrophil dysfunction thus hinders the dissociation of uterine epithelial-fetal membranes in parturient cows [38]. Additionally, hypocalcemia weakens uterine smooth muscle contractions [29,36], collectively increasing the incidence of RFM in dairy cows.

In addition, NEB in dairy cows, characterized by increased adipose mobilization and muscle degradation, results in the excessive production of highly oxidizing lipid peroxidation metabolites such as thiobarbituric acid and conjugated dienes, as well as ROS [39]. ROS accumulation induced by NEB is a primary driver of oxidative stress, and a positive correlation between the severity of NEB and the magnitude of oxidative stress in dairy cows has been well-documented [40,41]. RFM dairy cows exhibit exacerbated NEB and more severe oxidative stress, leading to disruptions in antioxidant defense mechanisms [24]. Consistent with these findings, the results of the present study demonstrate that periparturient RFM dairy cows display significantly reduced plasma activities of key antioxidant enzymes, specifically GSH-Px and CAT, accompanied by a marked elevation in the level of MDA, a stable marker of lipid peroxidation. These observations provide compelling evidence for the heightened oxidative stress status in RFM dairy cows. The pathophysiological consequences of oxidative stress in dairy cows extend beyond mere metabolic imbalance. Oxidative stress imposes a substantial burden on hepatic metabolic function, disrupts the metabolic homeostasis of the uterus, and exerts deleterious effects on critical cellular processes such as proliferation and apoptosis within uterine and placental tissues. These perturbations impair placental maturation and detachment, significantly elevating RFM susceptibility [22,42,43]. Collectively, the above findings underscore the intricate interplay between NEB, hypocalcemia, oxidative stress, and RFM pathogenesis, highlighting the need for targeted interventions to mitigate oxidative stress and NEB in dairy cows.

Using metabolomics approaches, we systematically characterized the metabolic imbalance features in RFM dairy cows during the prepartum period and also analyzed the association between this metabolic remodeling and RFM occurrence. In normal cows, metabolic function during the prepartum period exhibits stage-specific characteristics. At 21 days prepartum, cows primarily rely on dietary intake for metabolic energy, with amino acids preferentially allocated to mammary gland repair and fetal development. Oxidative stress levels remain low, and hepatic load is manageable [44,45]. By 7 days prepartum, dairy cows initiate a metabolic transition toward NEB, marked by enhanced gluconeogenesis, increased lipid mobilization, elevated antioxidant demand, and heightened hepatic load [44,46]. At parturition, metabolic energy demand surges, NEB intensifies, and further mobilization of adipose and muscle tissues occurs, accompanied by oxidative stress and potential liver dysfunction [23,47,48]. These metabolic adaptations reflect dynamic adjustments to support fetal development, parturition, and lactation initiation [49]. In contrast, the plasma metabolomics results of this study demonstrate that RFM cows exhibit metabolic alterations associated with negative energy balance as early as 21 days pre-calving, characterized by significant perturbations in adipose mobilization, muscle catabolism, and nucleotide metabolism. These findings provide valuable insights into clarifying the metabolic pathogenesis of RFM.

As to adipose mobilization, DMs in plasma of RFM dairy cows were closely associated with lipid metabolism pathways, including glycerophospholipid metabolism, GPI-anchor biosynthesis, and so on, suggesting early energy supply imbalance and altered lipid metabolism as early as 21 days prepartum [50,51]. Further functional analysis revealed that these differential lipid metabolites were primarily phospholipids involved in cell membrane remodeling, indicating enhanced membrane phospholipid remodeling and tissue mobilization in RFM cows [52,53,54]. Before calving, RFM dairy cows showed a pronounced decrease in plasma phosphatidylcholines, essential phospholipids produced by the liver for membrane synthesis, alongside a significant rise in lysophosphatidylcholines, which are degradation products of membrane breakdown. This reciprocal shift in lipid profiles indicates excessive mobilization of adipose and muscle reserves and impaired cellular membrane repair and turnover, likely due to disrupted phosphatidylcholine homeostasis [55].

Plasma metabolomics analysis further revealed that RFM cows exhibit amino acid metabolic disorders in the early peripartum period, characterized by significant perturbations in glycine-serine-threonine metabolic pathways and lysine degradation pathways. These alterations indicate premature muscle tissue mobilization and impaired amino acid homeostasis [56,57]. Regarding changes in differential metabolite concentrations, RFM cows showed signs of insufficient metabolic energy as early as 21 days prepartum. Muscle tissue mobilization was significantly enhanced at 7 days prepartum, while amino acid metabolite levels at 4 h postpartum indicated reduced muscle mobilization in RFM cows. These findings suggest that prepartum early-onset NEB leads to premature depletion of stored energy reserves, thereby compromising metabolic energy supply during parturition and impeding the progression of natural delivery [58].

NEB-induced tissue mobilization releases cellular DNA/RNA degradation products into the circulation, with plasma nucleotide metabolite levels reflecting tissue catabolism to some extent [59]. Concurrently, dysregulation of amino acid metabolic pathways (e.g., glycine and glutamate metabolism) disrupts nucleotide metabolic homeostasis [60]. The consistent concentration trends between nucleotide-related and amino acid differential metabolites further confirm the premature onset of NEB in RFM dairy cows. In summary, RFM dairy cows develop premature NEB by 21 days prepartum, accompanied by early adipose and muscle mobilization, disrupted membrane phospholipid remodeling, and amino acid metabolic dysregulation. By 7 days prepartum, elevated nucleotide metabolites indicate exacerbated tissue damage. This persistent metabolic dysfunction depletes energy reserves, impairs parturition adaptability, and constitutes a high-risk metabolic phenotype for RFM.

Both plasma biochemical indices and metabolomic profiles revealed that RFM cows exhibited NEB metabolic characteristics as early as 21 days prepartum, with the severity escalating toward parturition. In perinatal cows, NEB triggers lipid mobilization, leading to lipid deposition and structural abnormalities in placental tissues [61], thereby disrupting placental nutrient exchange and endocrine functions [62]. Concurrently, severe NEB and subsequent oxidative stress in RFM dairy cows perturb energy metabolism homeostasis in placental tissues, reduce uterine muscle tone, and adversely affect placental immune equilibrium [63]. These findings underscore the intricate link between systemic metabolic homeostasis and placental tissue metabolism, as well as other physiological functions.

To comprehensively characterize metabolic reprogramming in RFM cows during the perinatal period, we analyzed the small-molecule metabolite composition of postpartum placental and fetal membrane tissues. The results demonstrated that systemic metabolic disturbances in RFM cows significantly altered placental and fetal membrane metabolic functions. Significant changes occurred in the placenta and fetal membranes of RFM cows involving lipid metabolism (e.g., glycerophospholipid metabolism), amino acid metabolism (e.g., ABC transporters, phenylalanine, lysine, and glycine metabolism), nucleotide metabolism, and energy metabolism (e.g., oxidative phosphorylation and TCA cycle). These aberrant pathways indicate profound metabolic dysregulation in RFM placental tissues [27,64]. Given the high metabolic demands of parturition, prepartum metabolic dysfunction in RFM dairy cows predisposes them to energy deficiency during delivery [50,51].

Plasma metabolomics at 4 h postpartum revealed that normal cows exhibited higher mobilization efficiency of adipose and muscle tissues during parturition, consistent with placental and fetal membrane metabolomic findings. The levels of DMs associated with adipose/muscle tissue mobilization and nucleotide metabolism were significantly reduced in the placenta and fetal membranes of RFM cows. Under conditions of NEB, the variations in these metabolites primarily arise from differential mobilization of adipose and muscle tissues, and these metabolites mainly serve as key substrates in biological oxidation and energy production processes [65,66]. And so, this metabolic insufficiency in RFM placental and fetal membranes critically impairs energy supply during parturition, elevating RFM risk. The metabolome also highlighted associations between RFM and immune dysfunction [67,68,69], as well as extracellular matrix degradation at the fetal membrane-endometrium interface [70,71]. Similarly, aldosterone synthesis and secretion pathway dysregulation suggests endocrine disturbances could contribute to RFM by impairing uterine contractions [72]. Placental and fetal membrane metabolomics demonstrate that severe NEB-induced energy deficiency is a pivotal factor in RFM pathogenesis, while compromised placental immune function and dysregulated extracellular matrix degradation further exacerbate RFM susceptibility.

## 5. Conclusions

In summary, this study demonstrates that RFM in dairy cows is intrinsically linked to systemic metabolic dysregulation, characterized by premature and exacerbated NEB as early as 21 days prepartum. RFM dairy cows exhibit intensified adipose/muscle mobilization, disrupted phospholipid remodeling, amino acid imbalance, and elevated oxidative stress, collectively depleting energy reserves and impairing hepatic and placental functions. Metabolomic profiling reveals stage-specific perturbations in lipid metabolism (e.g., glycerophospholipid pathways), nucleotide catabolism, and energy-critical pathways (e.g., TCA cycle), culminating in insufficient bioenergetic supply during parturition. Concurrently, oxidative stress and immune-endocrine dysregulation further compromise placental detachment by disrupting extracellular matrix homeostasis and uterine contractility. These findings underscore RFM as a multifactorial syndrome rooted in periparturient metabolic insufficiency, highlighting the need for targeted nutritional or therapeutic strategies to mitigate prepartum NEB and oxidative stress, thereby reducing RFM incidence and improving postpartum health.

However, the present experimental data only demonstrate that RFM dairy cows experienced NEB 21 days before calving, confirming that early-onset NEB in the prepartum period is a critical inducer of RFM. The specific molecular regulatory mechanisms underlying this phenomenon remain to be elucidated. Additionally, further research should focus on developing peripartum cow TMR with optimized energy composition and palatability. Experimental studies are needed to evaluate its effects on improving energy metabolism in peripartum cows and to validate its potential for reducing RFM incidence.

## Figures and Tables

**Table 1:** Dietary ingredients and chemical composition of dairy cows during the perinatal period (dry matter basis).

Items ^1^	Ingredients and Chemical Composition
Ingredient	
Corn silage	38.72
Oat hay	24.34
Beet pulp	6.49
Rolled corn	4.06
Soybean meal	22.71
Yeast	0.16
Choline chloride	0.28
Vitamin-mineral mix	3.24
Nutrient composition	
DM, %	48.0
NEL ^2^, Mcal/kg DM	1.5
CP, % of DM	15.8
Starch, % of DM	15.0
NDF, % of DM	36.2
ADF, % of DM	21.9

^1^ DM = dry matter; NEL = net energy for lactation; CP = crude protein; NDF = neutral detergent fiber; ADF = acid detergent fiber. ^2^ Net energy for lactation was calculated according to NRC (2021).

## Data Availability

The authors confirm that all data underlying the findings are fully available without restriction.

## Supplementary Materials

The following supporting information can be downloaded at: https://www.mdpi.com/article/10.3390/ani16020229/s1, Table S1: Metabolomic data of plasma, placenta, and fetal membranes.

## Abbreviations

The following abbreviations are used in this manuscript:

RFM	retained fetal membranes
NEB	negative energy balance
DMI	dry matter intake
GLU	glucose
NEFA	non-esterified fatty acid
βHB	β-hydroxybutyric acid
LAC	lactic acid
LDH	lactic dehydrogenase
AST	aspartate aminotransferase
ALT	alanine aminotransferase
CK	creatine kinase
BUN	blood urea nitrogen
TBIL	total bilirubin
SOD	superoxide dismutase
GSH-Px	glutathione peroxidase
CAT	catalase
MDA	malondialdehyde
PLS-DA	Partial Least Squares Discriminant Analysis
DMs	differential metabolites
KEGG	Kyoto Encyclopedia of Genes and Genomes
Gpcho	phosphatidylcholines
Pc	Phosphatidylcholine
Ps	Phosphatidylserine
Dg	diacylglycerols
